# An Investigation of Surface EMG Shorts-Derived Training Load during Treadmill Running

**DOI:** 10.3390/s23156998

**Published:** 2023-08-07

**Authors:** Kurtis Ashcroft, Tony Robinson, Joan Condell, Victoria Penpraze, Andrew White, Stephen P. Bird

**Affiliations:** 1 Faculty of Computing, Engineering and the Built Environment, Ulster University, Derry BT48 7JL, UK; t.robinson@ulster.ac.uk (T.R.); j.condell@ulster.ac.uk (J.C.); 2 School of Cardiovascular and Metabolic Health, College of Medical, Veterinary and Life Sciences, University of Glasgow, Glasgow G12 8QQ, UK; victoria.penpraze@glasgow.ac.uk (V.P.); andrew.white@glasgow.ac.uk (A.W.); 3 School of Health and Medical Sciences, University of Southern Queensland, Ipswich, QLD 4305, Australia; stephen.bird@usq.edu.au

**Keywords:** surface electromyography, textile sEMG, oxygen consumption, internal load, external load, training load, compression shorts

## Abstract

The purpose of this study was two-fold: (1) to determine the sensitivity of the sEMG shorts-derived training load (sEMG-TL) during different running speeds; and (2) to investigate the relationship between the oxygen consumption, heart rate (HR), rating of perceived exertion (RPE), accelerometry-based PlayerLoad^TM^ (PL), and sEMG-TL during a running maximum oxygen uptake ($\dot{V}$O_2max_) test. The study investigated ten healthy participants. On day one, participants performed a three-speed treadmill test at 8, 10, and 12 km·h^−1^ for 2 min at each speed. On day two, participants performed a $\dot{V}$O_2max_ test. Analysis of variance found significant differences in sEMG-TL at all three speeds (*p* < 0.05). A significantly weak positive relationship between sEMG-TL and %$\dot{V}$O_2max_ (*r* = 0.31, *p* < 0.05) was established, while significantly strong relationships for 8 out of 10 participants at the individual level (*r* = 0.72–0.97, *p* < 0.05) were found. Meanwhile, the accelerometry PL was not significantly related to %$\dot{V}$O_2max_ (*p* > 0.05) and only demonstrated significant correlations in 3 out of 10 participants at the individual level. Therefore, the sEMG shorts-derived training load was sensitive in detecting a work rate difference of at least 2 km·h^−1^. sEMG-TL may be an acceptable metric for the measurement of internal loads and could potentially be used as a surrogate for oxygen consumption.

## 1. Introduction

Monitoring of the training load (TL) is an integral part of the training process in numerous sports and exercise regimes. The TL informs coaches of the physical and psychological stressors imposed on athletes during training and is categorised into two theoretical constructs: internal and external [1]. The internal TL captures the individual response to a given training stimulus and consists of objective physiological measures such as the heart rate (HR) or subjective psychophysiological methods such as the session rating of perceived exertion (sRPE) [2]. In contrast, the external load is the physical work completed by the individual. It includes measures such as global positioning systems (GPS), inertial sensors, time motion analysis (TMA), and local positioning systems (LPS) [3,4,5]. Combining the external TL with the internal response, or physiological changes in response to the load, is known as the dose–response relationship and is considered an important component in assessing the effectiveness of the training process [6]. It is suggested that adequate TL prescription could mitigate the chances of soft tissue injury, reduce illness, and increase game readiness [7,8].

It is recommended that a variety of internal and external load metrics are captured simultaneously as they are usually not interchangeable [9]. For example, HR-based measures of internal load demonstrate moderate–strong positive relationships with GPS metrics, such as jogging and total distance (*r* = 0.66), but no other GPS metrics, such as high-intensity running, sprinting, and maximum speed [4]. Contextual factors such as the upcoming opponent and previous game result (win, loss, and draw) can impact subjective TL methods, such as the RPE and sRPE, due to the psychological element that they inherit [4]. Thus, other objective internal TL monitoring approaches are recommended to capture different parameters of the individual response to sports and exercise.

Surface electromyography (sEMG) is a useful method for the evaluation of muscle activity, particularly in dynamic exercise. It has gained extensive usage in studying the electrophysiological aspects of muscle contractions and force generation within the realms of sports and exercise medicine [10]. Normally, the connection of wires to diagnostic equipment restricts free movement, which is a limiting factor in exercise-based sEMG protocols. The integration of sEMG sensors into clothing fabric, also referred to as “textile sEMG electrodes” [11,12,13,14], is potentially a novel technique for the objective measurement of the sEMG-derived training load (sEMG-TL). This would offer a convenient solution to the use of sEMG during dynamic exercise or sports activities in the field [14]. Strive™, Athos™, and Myontec™ provide wearable sEMG solutions claiming to capture sEMG-TL during exercise. Athos™ defines sEMG-TL as the total muscle activation from all sensors, divided by a scaling factor. While promising results exist for textile sEMG sensors in capturing muscular responses during isokinetic and functional exercise [11,14,15,16], research on sEMG-TL during dynamic exercise is limited.

Previous research has shown the strong validity and reliability of textile sEMG sensors embedded into compression shorts compared to a gold-standard BIOPAC system during knee flexion and extension, performed at different intensities [15]. Current research has used textile sEMG sensors to study muscle activity during controlled, closed-chain exercise [15,17], cycling [16], low-intensity daily locomotive activity [18], and functional exercise [11,14]. With claims that wearable sEMG shorts can be used in sports and exercise to provide a measure of TL, more research is warranted for assessing the sEMG derived TL metric given by the shorts during exercise.

The association between the integrated EMG (iEMG) of the quadriceps, hamstrings, and calf muscles and oxygen consumption suggests that muscle activation may be a representation of individuals’ physical exertion [19,20,21]. Thus, sEMG-TL may serve as a possible surrogate for oxygen consumption during specific treadmill running protocols, such as a $\dot{V}$O_2max_ test. To date, one study has demonstrated how sEMG shorts may be used as a viable predictor of lactate threshold work rates during an incremental cycling protocol [16]. This demonstrates the versatility of the use of sEMG shorts during exercise and they may be a viable tool in capturing the TL during dynamic whole-body exercise, such as running.

Catapult^TM^ utilises a triaxial accelerometer to calculate the PlayerLoad^TM^ (PL), an external load variable expressed as the square root of the sum of squared instantaneous acceleration changes in X, Y, and Z vectors, which has been validated in various sports and exercise research protocols [22,23].

External loads, such as PL, are often combined with internal loads such as HR measures to understand how prescribed training loads relate to individual athlete responses [24,25]. However, as previous research shows that accelerometry’s energy expenditure predictions decrease as the slope of uphill running increases due to inertial sensors’ limitations in detecting ground slope changes, a weak relationship between PL and oxygen consumption would be expected [26]. In contrast, sEMG-TL may better account for the changes in running slope during a treadmill running test as it accounts for the internal response.

Therefore, this study aims to (1) investigate the sensitivity of sEMG-TL across different running speeds; (2) explore and compare the relationships of sEMG-TL, HR, RPE, and PL with oxygen consumption during a maximal running test; and (3) examine the associations of sEMG-TL with HR, RPE, and PL. Understanding these relationships will contribute to advancing future research in the field of sports and exercise TL monitoring. Moreover, the findings will have practical implications, guiding the potential application of sEMG shorts as a tool for the monitoring of training loads in sports and exercise settings.

## 2. Materials and Methods

### 2.1. Participants

Six healthy, recreationally active males and four healthy, recreationally active females volunteered to participate in this study (Table 1). Written informed consent was obtained from all subjects involved in the study following a full explanation of the study’s research objectives, procedures, risks, and benefits. Ethical approval was obtained from the University of Glasgow Institutional Ethics Committee, and data collection procedures were in accordance with the Declaration of Helsinki.

### 2.2. Experimental Design 

Participants visited the laboratory on two consecutive days. On the first day, they undertook preliminary anthropometric tests, including body fat percentage recording using an air displacement plethysmograph (BodPod^®^, COSMED, Rome, Italy). Recording of the body fat percentage was deemed necessary as excess subcutaneous fat has been shown to weaken sEMG signals and dampen the peak amplitude [27]. The participants’ thigh length and hip circumference were measured to appropriately fit them with sEMG shorts (Athos^TM^, Redwood City, CA, USA). A neoprene vest that involved holding a triaxial accelerometer unit (OptimEye™ S5, Catapult Innovations, Melbourne, Australia) between the scapulae was also fitted to each participant. Participants were familiarised with the wearable technology and a stationary sEMG calibration protocol, which was undertaken to configure the sEMG thresholds for each muscle group. The sEMG sensors were positioned to measure four muscle groups on each leg (inner and outer quadriceps, hamstrings, and glutes). Following the calibration protocol, participants completed a 3-speed treadmill running test. This test was conducted to assess whether the sEMG-TL could detect small changes in workload, as, to date, no research has explored the sEMG-TL. 

On the second day, participants completed a treadmill running maximum oxygen uptake ($\dot{V}$O_2max_) test, during which various internal loads (sEMG-TL, RPE, HR, $\dot{V}$O_2_) and external loads (accelerometery) were continuously monitored. 

### 2.3. Methodology 

**Figure 1:**
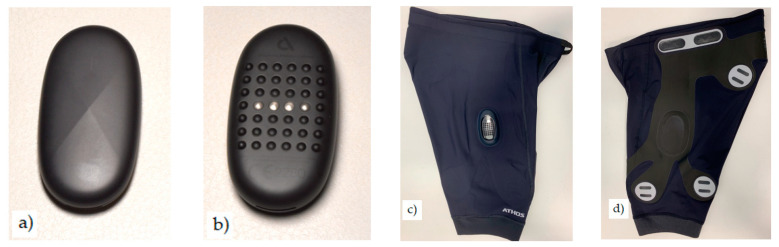
Athos^TM^ unit anterior view (**a**) and posterior view (set of contacts) (**b**). Exterior right leg (**c**) and interior left leg (**d**) view of sEMG shorts. Note: sEMG dry electrodes and electrode leads are composed of an inkjet-printed conductive polymer comprising an ether-based conductive thermoplastic polyurethane material. The electrodes are overlaid with a soft conductive silicone, which increases the stability of the electrode–skin interface.

**Figure 2:**
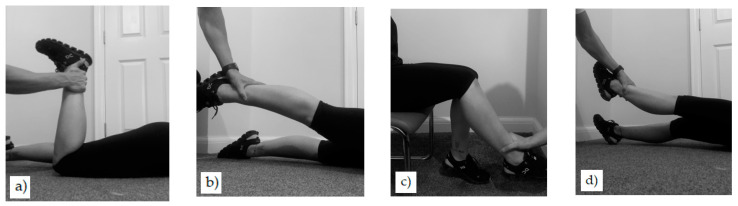
Movements for the sEMG calibration protocol to establish sEMG amplitude thresholds. Movements include prone knee flexion (**a**), prone hip extension (**b**), seated knee extension (**c**), and supine leg raise (**d**).

On the first day, participants wore the sEMG shorts and were allocated an Athos unit (Figure 1) and completed the sEMG calibration protocol, which included four specific movements: seated leg extension, supine leg raise, prone knee flexion, and prone hip extension. Each movement was repeated four times while the researcher manually applied varying passive forces (low, medium, and high) and provided resistance to induce an isometric maximal voluntary contraction (MVC) (Figure 2). Following the sEMG calibration, each participant was fitted with a Polar H10 heart rate chest monitor (Polar Electro Oy, Kempele, Finland) and a triaxial accelerometer. Participants were then required to undertake a 5-min jogging warm-up on a treadmill (Woodway, ELG 70 Weiss, Weil am Rhein, Germany). The warm-up was important to increase the stability of the silicone overlaid electrode-to-skin interface, and to promote perspiration to increase skin conductivity. The 3-speed treadmill test then commenced, which required the participants to run at 8 (low), 10 (mod), and 12 (high) km⋅h^−1^ for 2 min at each speed. 

On the second day, participants completed an incremental treadmill exercise test to determine $\dot{V}$O_2max_. Males ran at 10 km⋅h^−1^, while females ran at 8 km⋅h^−1^, both on a 1% gradient increase every 1 min until reaching volitional exhaustion. Breath-by-breath pulmonary gas exchange was measured using a metabolic cart (MedGraphicsTM, Gloucester, UK) to measure $\dot{V}$O_2_ (mL·kg^−1^·min^−1^), which was averaged over consecutive 60 s intervals. At the end of each 60 s interval, the participants’ heart rate (bpm) and RPE (Borg’s Category Ratio 6–20 RPE scale) were also measured.

### 2.4. Data Collection and Processing

The Athos™ unit electronics subsystem is responsible for signal reception, transmission, and conditioning. Within the electronics subsystem is a signal conditioning module that filters the sEMG data. Sampled sEMG signals were captured at 1 kHz. The anti-aliasing filter applied prior to sampling prevented high-frequency noise greater than 500 Hz from aliasing into the sEMG spectrum. Filtering included a linear band-pass with a cut-off frequency from 10 Hz to 500 Hz (23 dB frequencies with centre frequency at 120 Hz); a notch (removal of 60 Hz noise) filter, rectification, and linear envelope were applied. The linear envelope was down-sampled by a factor of 25 and smoothed using a 16-sample root-mean-square (RMS) transformation along with signal conversion from analogue to digital. sEMG signals were averaged at a 10 s window. sEMG recordings were considered viable when impedance was below 5 kΩ. Poor contact quality was deemed if loss of the contact signal occurred over 10% of the time. These filtering and signal processes were predetermined by the Athos™.

The sEMG peak amplitude, which was captured through the calibration protocol, provided individualised reference points for each muscle group, which the raw integrated sEMG output (area under the curve of the rectified EMG signal) could be normalised against. The integrated sEMG for each muscle group was measured as a percentage of the MVC. The accumulation of the normalised integrated sEMG values across all muscle groups relative to the MVC peak sEMG amplitude represented the sEMG-TL reported in arbitrary units (a.u.). A single arbitrary unit was equivalent to one muscle activating at 100% of the MVC for 1-s.

The triaxial accelerometer sampled at 100 Hz. The data were accumulated to generate a measure of the external load (PL). The equation for PL can be seen below:

(1) $$\mathrm{PL}=\sqrt{{\left(a_{x_{i}}-a_{x_{i-1}}\right)}^{2}+{\left(a_{y_{i}}-a_{y_{i-1}}\right)}^{2}+{\left(a_{z_{i}}-a_{z_{i-1}}\right)}^{2}}$$

where $a_{x}$ is the mediolateral acceleration, $a_{y}$ the anteroposterior acceleration, and $a_{z}$ the vertical acceleration.

### 2.5. Statistical Analysis 

The data were analysed using the statistical software package SPSS (version 26.0). Since we had a small sample size, determination of the distribution of the data for each variable (sEMG-TL, HR, RPE, PL, and %$\dot{V}$O_2max_) using a Shapiro–Wilk test was performed. Results showed that the distributions for all variables (sEMG-TL, HR, RPE, PL, and %VO_2max_) departed significantly from normality (W = 0.92–0.96, *p* < 0.05), as well as the 3-speed test data (W = 0.82, *p* < 0.05). Descriptive statistics were determined for the participants’ sEMG-TL data from the 3-speed test and for all variables during each minute in the $\dot{V}$O_2max_ test. The sensitivity of the sEMG-TL in detecting 2 km⋅h^−1^ changes in running intensity during the 3-speed treadmill test was assessed by a Kruskal–Wallis one-way analysis of variance by ranks test, and pairwise comparisons were performed using a Dunn–Bonferroni post hoc test. Statistical significance was set at *p* < 0.05. Raw accelerometry data and $\dot{V}$O_2_ (mL·kg^−1^·min^−1^) were averaged at every 60 s interval during the $\dot{V}$O_2max_ test. $\dot{V}$O_2_ was normalised to $\dot{V}$O_2max_ (%$\dot{V}$O_2max_). Thus, a non-parametric Spearman’s correlation test was applied to determine the correlations between all variables in the composite data set, while separate correlations were applied on the individual data sets for sEMG-TL, HR, RPE, PL, and %$\dot{V}$O_2max_. The significance level was set at *p* < 0.05. Correlations were classified as previously suggested: 0 = no correlation, (0 < r_s_ < 0.2) = very weak correlation, (0.2 ≤ r_s_ < 0.4) = weak correlation, (0.4 ≤ r_s_ < 0.6) = moderate correlation, (0.6 ≤ r_s_ < 0.8) = strong correlation, (0.8 ≤ r_s_ < 1.0) = very strong correlation, and 1 = perfect correlation [28].

## 3. Results

### 3.1. Three-Speed Treadmill Test

**Figure 3:**
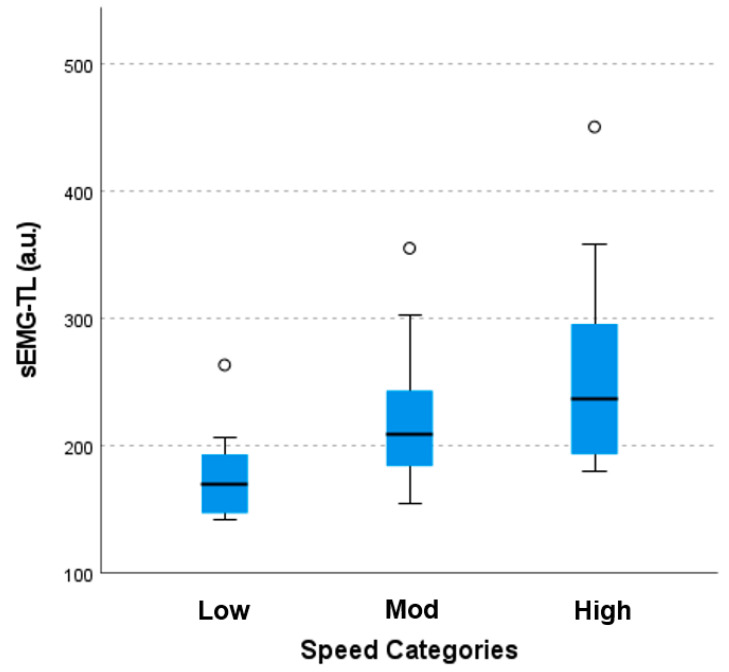
Boxplot showing the distribution of sEMG-TL across different running speeds. sEMG-TL = surface electromyography training Load; a.u. = arbitrary units; low = 8 km⋅h^−1^; mod = 10 km⋅h^−1^; high = 12 km⋅h^−1^; black line = median, and black dots = 1 participant with a very high sEMG-TL.

sEMG-TL is presented as a median (interquartile range (IQR)) for each running speed. Figure 3 illustrates the spread of data between each running speed. A low running speed resulted in the lowest median sEMG-TL, 254.17 a.u. (IQR = 148.50 to 204.27 a.u.). A high running speed resulted in the highest sEMG-TL, 236.67 a.u. (IQR = 192.10 to 295.48 a.u.), and the sEMG-TL for a moderate running speed was between those of the low and high speeds, 208.07 (IQR = 183.51 to 244.29 a.u.). The Kruskal–Wallis one-way analysis of variance by ranks test revealed a significant difference in sEMG-TL among the three running speeds, *χ*^2^(2) = 11.50, *p* < 0.05. Post hoc pairwise comparisons using the Dunn–Bonferroni test demonstrated that the sEMG-TL at a low running speed was significantly different from both the moderate and high running speeds (adjusted *p* < 0.05), but no statistical difference was established between moderate and high running speeds (adjusted *p* > 0.05).

### 3.2. Treadmill $\dot{V}$O_2max_ Test

There were 74 individual recordings included in the analysis for each dependent variable. Median (IQR) loads are presented in Table 2 for each measurement every 1 min during the $\dot{V}$O_2max_ test. Generally, the median load for each variable increased at every 1 min interval during the $\dot{V}$O_2max_ test. 

**Figure 4:**
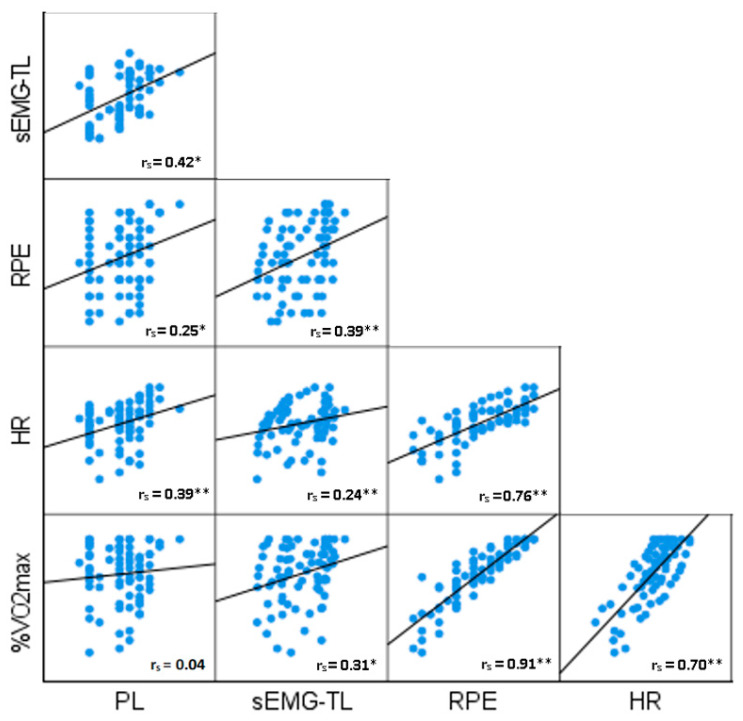
Scatterplot matrix of associations between variables: %$\dot{V}$O_2max_ = percentage of maximum oxygen uptake; sEMG-TL = surface electromyography training load; RPE = rating of perceived exertion; HR = heart rate; PL = PlayerLoad. Each blue dot corresponds to individual measurements at each one-minute stage during the treadmill running test. Solid lines are the least-squares derived best-fitting lines. * *p* < 0.05; ** *p* < 0.01.

Figure 4 presents illustrates the Spearman’s rank correlation coefficients between each load variable for the composite data set. While HR and RPE showed significant strong positive correlations (r_s_ = 0.70, *p* = 0.002) and r_s_ = 0.91, *p* = 0.001, respectively) with %$\dot{V}$O_2max_, sEMG-TL revealed a very weak correlation (r_s_ = 0.31, *p* = 0.02). In contrast, no correlation was established between PL and %$\dot{V}$O_2max_ (r_s_ = 0.04, *p* = 0.74). sEMG-TL showed weak significant correlations between RPE (r_s_ = 0.39, *p* = 0.01), HR (r_s_ = 0.24, *p* = 0.03) and PL (r_s_ = 0.42, *p* = 0.02).

Correlation coefficients at the individual level are presented in Table 3. Most notably, sEMG-TL demonstrated very strong significant positive correlations with %$\dot{V}$O_2max_ in nine out of ten participants, while only three participants showed very strong non-significant positive correlations between PL and %$\dot{V}$O_2max_. In addition, Table 3 presents very strong significant positive correlations between RPE and sEMG-TL in seven out of ten participants, while three participants showed non-significant strong correlations. Similarly, HR and sEMG-TL were very strongly significantly correlated in eight out of ten participants, while two participants showed strong non-significant positive correlations. 

## 4. Discussion

The purpose of this study was to (1) investigate the sensitivity of sEMG-TL across different running speeds; (2) explore and compare the relationships of sEMG-TL, HR, RPE, and PL with oxygen consumption during a maximal running test; and (3) examine the associations of sEMG-TL with HR, RPE, and PL. An important feature of this study is the novelty of the textile sEMG sensors embedded in compression shorts to provide an sEMG-based internal TL metric as a method for the objective monitoring of the muscular load during dynamic exercise. The results show that sEMG-TL exhibited significant differences among three different running intensities, distinguished by increments of 2 km⋅h^−1^ in running velocity. As expected, higher running velocities were associated with an increased sEMG-TL. Figure 3 illustrates the distribution of participants’ sEMG-TL for each speed. A significant difference was shown between low–moderate and low–high, but, surprisingly, not moderate–high speeds. While the sEMG-TL was higher, the larger spread of the data was likely attributed to a non-significant difference. In fact, the spread of the data was greater as the speed increased. This may imply that the sEMG sensors become less accurate at higher intensities. However, it is important to also consider the individual biomechanical and physiological factors that could result in interindividual differences that affect the sEMG signal. Overall, these findings reflect the increased excitability of the muscles in response to the escalating demands imposed by running at higher velocities. Previous findings support these findings, showing increased sEMG signal amplitudes in running compared to low-speed jogging [29]. These findings indicate that sEMG-TL is sensitive in detecting small changes in intensity during exercise. 

Considering the second aim of the study, a significant yet somewhat modest association between sEMG-TL and %$\dot{V}$O_2max_ was established (Figure 4). However, at the individual level, very strong significant correlations between sEMG-TL and %$\dot{V}$O_2max_ were established in ninety percent of the participants (Table 3). The interplay of participants’ unique biomechanical and physiological characteristics likely resulted in a weaker relationship between sEMG-TL and %$\dot{V}$O_2max_ in the pooled data set. Previous research identified that when analysing grouped data, the median frequency values of integrated EMG were insensitive to changes in muscle recruitment, metabolite accumulation, and fatigue associated with the increases in work intensity during a cycle ergometer $\dot{V}$O_2max_ test [30]. However, the integrated EMG results were highly individualistic between some subjects and muscles, which could help to explain the findings in the present study. Previous studies have also reported similar results regarding the intra-individual variability based on the sEMG signal amplitude [31,32]. During the $\dot{V}$O_2max_ test, the velocity of the treadmill was maintained at a constant level while the gradient of the treadmill increased by 1% every minute. As more force output is required every minute during the test, there is a higher demand for oxygen by the oxidative pathway, which metabolises to produce adenosine triphosphate (ATP) for energy. This was reflected through the $\dot{V}$O_2_ measured from the participant. The higher force output requirements and associated fatigue as the test progresses will stimulate the muscles to increase the electrical discharge to compensate for the decreasing muscle strength, resulting in an increased sEMG amplitude [33]. During fatiguing exercise, the more frequent recruitment of larger motor unit action potentials is necessary to sustain the required force output during the test, which increases the sEMG amplitudes and, in part, explains the association between sEMG-TL and %$\dot{V}$O_2max_ [33]. 

HR and RPE demonstrated very strong significant correlations with $\%\dot{V}$O_2max_ both in the composite data set and at the individual level for all participants. These findings align with the previous literature, suggesting that both HR and RPE are reliable indicators of intensity and load in the context of load monitoring [2,34,35]. It is important to emphasise that the load monitoring approaches employed in this study captured distinct parameters of the load from the participants, as exemplified through the varying degrees of correlation with $\%\dot{V}$O_2max_. HR, being a measure of the heart’s response to exercise, appears to be particularly effective in representing the oxygen output during physical activity, as anticipated, given its direct reflection of the cardiovascular system’s response to exertion. These results support the use of HR and RPE for load monitoring, as they highlight the utility of HR and RPE as practical measures to assess $\%\dot{V}$O_2max_ during exercise. By capturing complementary aspects of the participants’ physiological responses, HR and RPE provide valuable insights into the intensity of the exercise and are more strongly related to $\%\dot{V}$O_2max_ than sEMG-TL and PL.

PL demonstrated no correlation with $\%\dot{V}$O_2max_ for the grouped data. This highlights the different parameters that internal and external player monitoring systems capture during exercise [2,25,36]. The type of treadmill running test used in the present study may have negatively influenced the relationship between PL and $\%\dot{V}$O_2max_. It has been previously shown that sEMG from the quadriceps and hamstrings may better reflect energy expenditure during uphill and downhill locomotion compared to accelerometry [37]. The metabolic rate during running is determined by two factors: (a) the rate of muscle force development, such as during the stance phase in running, where the tensile properties of soft tissue produce force; and (b) the volume of active leg muscle [38,39]. Thus, as the gradient of the treadmill increases, the sEMG-TL may be a better representation of the load experienced by the individual as PL only captures acceleration. 

In regard to the third aim of the study, the current findings established a significant weak association between sEMG-TL and RPE in the composite data set (Figure 4). Previous research has illustrated similar results [40]. Fontes et al. found a moderate positive correlation between RPE and sEMG during exhaustive constant-load cycling bouts. Previous research has also indicated that the recruitment of additional muscle fibres is associated with increased RPE [41]. In addition, type II muscle fibre recruitment has been shown to increase the sEMG amplitude, which occurs at greater intensities nearing the end of the $\dot{V}$O_2max_ test, which in part may explain this relationship. At the individual level, sEMG-TL demonstrated very strong significant correlations with RPE in seventy percent of participants. Establishing a relationship between RPE and sEMG-TL is an important finding as this could help with exercise prescription and in designing routines for specific muscle loads.

In addition, a significant, very weak correlation was found between HR and sEMG-TL in the composite data, yet very strong significant correlations were established in eighty percent of participants when the HR and sEMG-TL correlations were analysed at the individual level. While there is no direct causal relationship between sEMG and HR, they are often positively associated during exercise [42,43]. As muscle activity increases, indicated by sEMG-TL, HR increases as well to meet the metabolic demands. 

### Strengths and Limitations

Most of the previous research conducted on textile sEMG sensors embedded in shorts for the monitoring of muscle activity involves isokinetic exercises, general locomotion, or low-level functional exercise [14,15,16,17,18]. In general, most studies investigate specific muscle groups’ sEMG responses, whereas this study used sEMG shorts to derive a TL metric, which opens new research possibilities in monitoring sEMG-TL in different sports and exercise environments. Additionally, sEMG-TL appears to closely correlate with $\%\dot{V}$O_2max_ at the individual level; thus, the shorts could serve as a potential surrogate for the measurement of $\dot{V}$O_2_ during incremental running. 

Individual biomechanical and physiological characteristics likely impact the relationship between sEMG-TL and the other load variables [30,31,32]. A limiting factor in the current research was the lack of control of contextual factors, such as the participants’ training experience, fitness level, running technique, and muscle fibre type composition. For example, previous research has shown that athletes who are fitter and inherit better technique recruit muscle at different rates compared to non-athletes, especially in higher threshold motor units [44]. Thus, ensuring sample homogeneity, such as participants with similar fitness characteristics, could reduce confounding variables and enhance the internal validity of the study. In addition, a weakness of this study was that it entailed a small sample size. A larger sample size could potentially enhance the statistical power and generalisability of the results. 

Another limitation of the current study is that, while HR and RPE are global measurements of internal load, sEMG represents a portion of superficial muscle (upper legs, only), yet the participants likely experienced physiological stress elsewhere in the body. Therefore, caution should be taken when using the data to inform exercise loading parameters where exercise includes more muscles other than the upper leg. In the future, research might consider using a full-body sEMG compression garment that encompasses many more sEMG sensors to better reflect the whole-body muscle load.

## 5. Conclusions

This study aimed to investigate the sensitivity of sEMG-TL across different running speeds, explore its relationship with oxygen consumption, and examine its associations with HR, RPE, and PL. The novelty of using textile sEMG sensors embedded in compression shorts provided an objective method for the monitoring of the muscular load during dynamic exercise. 

The results revealed significant differences in sEMG-TL among different running intensities, with higher velocities associated with increased sEMG-TL. The study also established a significant yet weak association between sEMG-TL and $\%\dot{V}$O_2max_ at the group level, but strong individual correlations were found. HR and RPE showed very strong significant correlations with $\%\dot{V}$O_2max_, supporting their reliability for the assessment of exercise loads. However, PL demonstrated no correlation with $\;\%\dot{V}$O_2max_, indicating the limitations of accelerometry in capturing loads during treadmill running with gradient increases in the uphill slope. Additionally, a significant weak association was found between sEMG-TL and RPE at the composite level, while showing more substantial individual-level correlations. The study’s strengths include the use of sEMG shorts to derive a TL metric and its potential application for individual exercise prescription. However, factors such as the small sample size and the need to control contextual factors were identified as study limitations. 

In conclusion, this research emphasises the sensitivity of sEMG-TL in detecting small changes in exercise intensity and highlights the potential for future research to be conducted with training load monitoring using sEMG. Future research should consider larger sample sizes and use full-body sEMG compression garments to better reflect the whole-body muscle load during exercise.

## Figures and Tables

**Table 1:** Participant characteristics.

Characteristic	Female	Male
Age (years)	23.5 ± 1.5	26.1 ± 3.7
Body mass (kg)	59.3 ± 3.6	78.3 ± 9.4
Height (m)	165.8 ± 8.0	180.4 ± 8.3
Body fat (%)	30.9 ± 6.3	17.4 ± 5.5
$\dot{V}$O_2max_ (mL⋅kg ^−1^⋅min^−1^)	40.8 ± 2.6	49.6 ± 6.0

Note: sEMG-TL = surface electromyography training load; RPE = rating of perceived exertion; HR = heart rate; PL = PlayerLoad. Values are presented as mean ± SD.

**Table 2:** Medians (IQR) for variables during each minute of the treadmill $\dot{V}$O_2max_ test.

No. of Participants	$\mathrm{Minute}\;\mathrm{of}\;\dot{V}$O_2max_	sEMG-TL (a.u)	HR (bpm)	RPE (a.u.)	PL (a.u.)
10	1	85.85 (63.77, 100.37)	138 (128, 156)	8 (7, 12)	15.00 (12.00, 16.75)
10	2	81.42 (69.40, 105.46)	166 (151, 170)	11 (9, 14)	15.00 (12.75, 16,25)
10	3	82.79 (71.40, 108.62)	163 (159, 178)	13 (11, 14)	15.50 (13.55, 16,25)
10	4	87.07 (71.96, 109.15)	167 (159, 176)	15 (11, 17)	15.50 (13.50, 16.25)
9	5	78.44 (72.76, 107.05)	170 (164, 181)	14 (12, 17)	16.00 (12.00, 17.00)
7	6	94.26 (72.30, 109.51)	177 (170, 194)	16 (15, 19)	16.00 (12.00, 17.00)
7	7	102.89 (74.31, 112.42)	181 (152, 192)	16 (9, 19)	16.00 (13.00, 17.00)
6	8	94.52 (68.57, 110.88)	182 (164, 186)	18 (13, 19)	16.50 (12.75, 18.75)
3	9	112.69 (94.35, 116.31)	186 (180, 189)	18 (17, 18)	17.00 (12.00, 18.50)
1	10	113.15	204	20	18.00
1	11	101.00	204	20	19.00

Note: sEMG-TL = surface electromyography training load; RPE = rating of perceived exertion; HR = heart rate; PL = PlayerLoad.

**Table 3:** Individual correlations between variables for each participant.

Participant		1	2	3	4	5	Participant	1	2	3	4	5
1	PL						2					
	sEMG-TL	0.33						−0.09				
	$\%\dot{V}$O_2max_	0.42	0.83 **					−0.57	0.80			
	RPE	0.46	0.81 **	0.98 **				−0.38	0.92 *	0.96 **		
	HR	0.48	0.78 *	0.98 **	0.97 **			−0.76	0.61	0.94 *	0.82	
3	PL						4					
	sEMG-TL	0.49						0.67				
	$\%\dot{V}$O_2max_	0.42	0.81 *					0.49	0.92 **			
	RPE	0.40	0.67	0.95 **				0.62	0.99 **	0.96 **		
	HR	0.43	0.84 **	0.99 **	0.95 **			0.38	0.81 *	0.96 **	0.87 **	
5	PL						6					
	sEMG-TL	−0.07						0.66 *				
	$\%\dot{V}$O_2max_	−0.16	0.90 **					0.85 **	0.84 **			
	RPE	−0.04	0.99 **	0.89 **				0.79 **	0.88 **	0.99 **		
	HR	−0.15	0.94 **	0.99 **	0.93 **			0.82 **	0.74 **	0.98 **	0.96 **	
7	PL						8					
	sEMG-TL	0.30						0.91 *				
	$\%\dot{V}$O_2max_	0.31	0.97 *					0.97 **	0.96 **			
	RPE	0.00	0.56	0.73				0.96 **	0.83	0.90 *		
	HR	0.34	0.95	0.99 **	0.77			0.95 *	0.99 **	0.99 **	0.88	
9	PL						10					
	sEMG-TL	−0.60						0.88 **				
	$\%\dot{V}$O_2max_	−0.83 *	0.85 **					0.74	0.88 **			
	RPE	−0.74 *	0.96 **	0.95 **				0.75	0.88 **	0.99 **		
	HR	−0.83 *	0.86 **	0.99 **	0.96 **			0.79 *	0.95 **	0.97 **	0.97 **	

Note: %$\dot{V}$O_2max_ = percentage of maximum oxygen uptake; sEMG-TL = surface electromyography training load; RPE = rating of perceived exertion; HR = heart rate; * *p* < 0.05; ** *p* < 0.01.

## Data Availability

The data generated in this study are not available in any public network database. For anyone who would like to have access to the data, please contact the author of the study, K.A., email: k.ashcroft@ulster.ac.uk.
